# Utidelone plus PD-1 Inhibitors as Later-Line Combination Treatment in Advanced Solid Tumors after Failure of Multiple Standard Therapies: A Case Series

**DOI:** 10.1159/cro/abzag051

**Published:** 2026-09-08

**Authors:** Lisha Wu, Huisheng Chen, Zihang Lin, Haiwei Xiao, Tao Qin

**Affiliations:** a Department of Oncology, Shenshan Medical Center, Sun Yat-sen Memorial Hospital, Sun Yat-sen University, Shanwei, China; b Department of Oncology, Sun Yat-sen Memorial Hospital, Sun Yat-sen University, Guangzhou, China; c Department of Traditional Chinese Medicine, Shenshan Medical Center, Sun Yat-sen Memorial Hospital, Sun Yat-sen University, Shanwei, China

**Keywords:** Utidelone, PD-1 inhibitors, Later-line treatment, Case series

## Abstract

**Introduction:**

The later-line treatment for advanced solid tumors that have progressed after multiple lines of therapy remains limited and challenging. Utidelone (UTD1), an analog of epothilone B, has shown significant antitumor activity in breast cancer undergone at least one previous chemotherapy in China. However, the combined therapeutic effect of UTD1 and programmed cell death protein-1 (PD-1) inhibitors in heavily pretreated solid tumors is unclear.

**Case Presentation:**

Herein, we report a case series of 3 patients with heavily pretreated advanced solid tumors, including nasopharyngeal carcinoma and pulmonary adenocarcinoma, who had progressed after multiple lines of standard therapy. Treatment with UTD1 in combination with PD-1 inhibitors demonstrated promising efficacy. Individual progression-free survival times were 9, 5, and 6 months, respectively, with 2 patients achieving a partial response and one achieving stable disease. Although peripheral neuropathy was a common adverse event, it was generally manageable.

**Conclusion:**

Our case series highlights that UTD1 plus PD-1 inhibitors could become a novel later-line treatment option for patients with advanced solid tumors that have failed multiple standard therapies.

## Introduction

Treatment of advanced solid tumors that have become refractory to multiple standard regimens remains a major clinical challenge. Epothilones are microtubule inhibitors produced through the metabolism of the cellulose-degrading myxobacterium *Sorangium cellulosum* [1], which exhibited similar anticancer properties with paclitaxel by disrupting the dynamic equilibrium of microtubules, thereby preventing tumor cell proliferation. Ixabepilone (Ixempra^®^), a semi-synthetic epothilone analog, is the only compound in its class granted approval by the US Food and Drug Administration (FDA) as a single-agent regimen for the treatment of metastatic or locally advanced breast cancer that has proven refractory to prior lines of therapy, including anthracyclines, taxanes, and capecitabine [2]. However, common toxicities related to ixabepilone, such as myelosuppression, hepatic toxicity, and peripheral neuropathy (PN), often lead to treatment discontinuation [2–4]. Additionally, the high costs associated with ixabepilone, as a result of several chemical synthesis steps after bacterial fermentation, significantly impede its clinical application [5, 6].

Utidelone (UTD1), a novel epothilone B derivative, is biosynthesized through targeted genetic modification of the polyketide synthase gene cluster in *S. cellulosum* [7]. The improved manufacturing approach enables cost-efficient large-scale production of UTD1. UTD1 has demonstrated significant antitumor activity in breast cancer. A phase III clinical trial revealed the significant efficacy of UTD1 in combination with capecitabine, which prolonged progression-free survival (PFS) and overall survival in patients with metastatic breast cancer who were unresponsive to anthracycline and taxane chemotherapy compared to those receiving capecitabine alone [8, 9]. Consequently, UTD1 has been approved by China National Medical Products Administration (NMPA) for the treatment of metastatic breast cancer patients, who have undergone at least one previous chemotherapy regimen. In addition to breast cancer, some preliminary cases indicate that UTD1 exhibited notable antitumor activity in other advanced or metastatic solid tumors that have failed standard treatment [10–12]. However, the exact combined therapeutic effect of UTD1 and PD-1 inhibitors in heavily pretreated advanced solid tumors remains unclear. Herein, we present a case series of 3 patients with heavily pretreated advanced solid tumors who had failed multiple lines of therapy. The administration of UTD1 combined with immune checkpoint inhibitor of the PD-1 demonstrated promising outcomes, providing an alternative later-line treatment in patients with heavily pretreated advanced tumors. The case series was prepared in accordance with the CARE reporting guidelines, and the completed CARE checklist is available as online supplementary material (for all online suppl. material, see https://doi.org/10.1159/cro/abzag051).

## Case Presentations

### Case 1

In May 2020, a 50-year-old Chinese woman with no previous history of other diseases was diagnosed with local advanced nasopharyngeal carcinoma, non-keratinizing squamous cell carcinoma, undifferentiated subtype. She initially received docetaxel, lobaplatin, and nimotuzumab as first-line chemotherapy, as well as subsequent radical radiotherapy, achieving 11-month PFS. In April 2021, follow-up examination revealed lung metastasis, and the patient received second-line treatment with tegafur combined with apatinib in a 21-day cycle, resulting in a PFS of approximately 12 months. In April 2022, this patient had extensive disease progression, including multiple bone and cervical lymph node metastasis. Third-line nab-paclitaxel plus carboplatin chemotherapy was given and the PFS was about 8 months. In December 2022, due to newly developed liver metastasis, gemcitabine combined with toripalimab (PD-1 inhibitor) was selected as the fourth-line treatment and also achieved 8-month PFS. In August 2023, both lung and liver metastasis progression occurred, and the patient started fifth-line treatment with vinorelbine alone every 3 weeks. Unfortunately, after two chemotherapy cycles, the patient presented lung and liver metastasis progression (Table 1).

**Table 1.** Clinical features and outcomes of 3 patients receiving UTD1 plus PD-1 inhibitors

Case	Diagnosis	Therapy line		Response	PFS, months
1	Nasopharyngeal carcinoma	6	1st line: docetaxel, iobaplatin, and nimotuzumab	PR	9
			2nd line: tegafur and apatinib		
			3rd line: nab-paclitaxel and carboplatin		
			4th line: gemcitabine and toripalimab		
			5th line: vinorelbine		
			6th line: UTD1 and camrelizumab		
2	Pulmonary adenocarcinoma	4	1st line: pemetrexed, carboplatin, sintilimab, and bevacizumab	PR	5
			2nd line: vinorelbine		
			3rd line: sintilimab and anlotinib		
			4th line: UTD1 and sintilimab		
3	Pulmonary adenocarcinoma	3	1st line: pemetrexed, carboplatin and sintilimab	SD	6
			2nd line: nab-paclitaxel and bevacizumab		
			3rd line: UTD1 and camrelizumab		

PFS, progression-free survival; PR, partial response; SD, stable disease.

In December 2023, the patient received sixth-line treatment with UTD1 (30 mg/m^2^ intravenously once per day on days 1–5) plus PD-1 inhibitor camrelizumab (200 mg intravenously once per day on day 1) of each cycle, once every 3 weeks. Surprisingly, after two cycles of treatment, respectively, the patient had improved performance status and achieved partial response (PR) according to Response Evaluation Criteria in Solid Tumors (RECIST) version 1.1. Her liver metastasis showed continuous PR while lung metastasis stable in the next six cycles (Fig. 1). The patient developed grade 2 PN, graded according to the Common Terminology Criteria for Adverse Events (CTCAE) version 5.0 [13], manifesting as numbness in the hands and feet, with symptom onset after approximately the sixth cycle. She received mecobalamin for neurologic adverse events. The dose was reduced by 20% starting from the eighth cycle. After nine cycles, the patient refused to continue UTD1 due to the PN. Her neuropathic symptoms resolved spontaneously after UTD1 discontinuation. She subsequently received metronomic capecitabine plus camrelizumab. After two cycles of this subsequent therapy, a follow-up CT scan revealed progressive disease in both the lung and liver metastases. The PFS from the initiation of UTD1 plus camrelizumab to confirmed progression was approximately 9 months (Table 1).

**Fig. 1.**
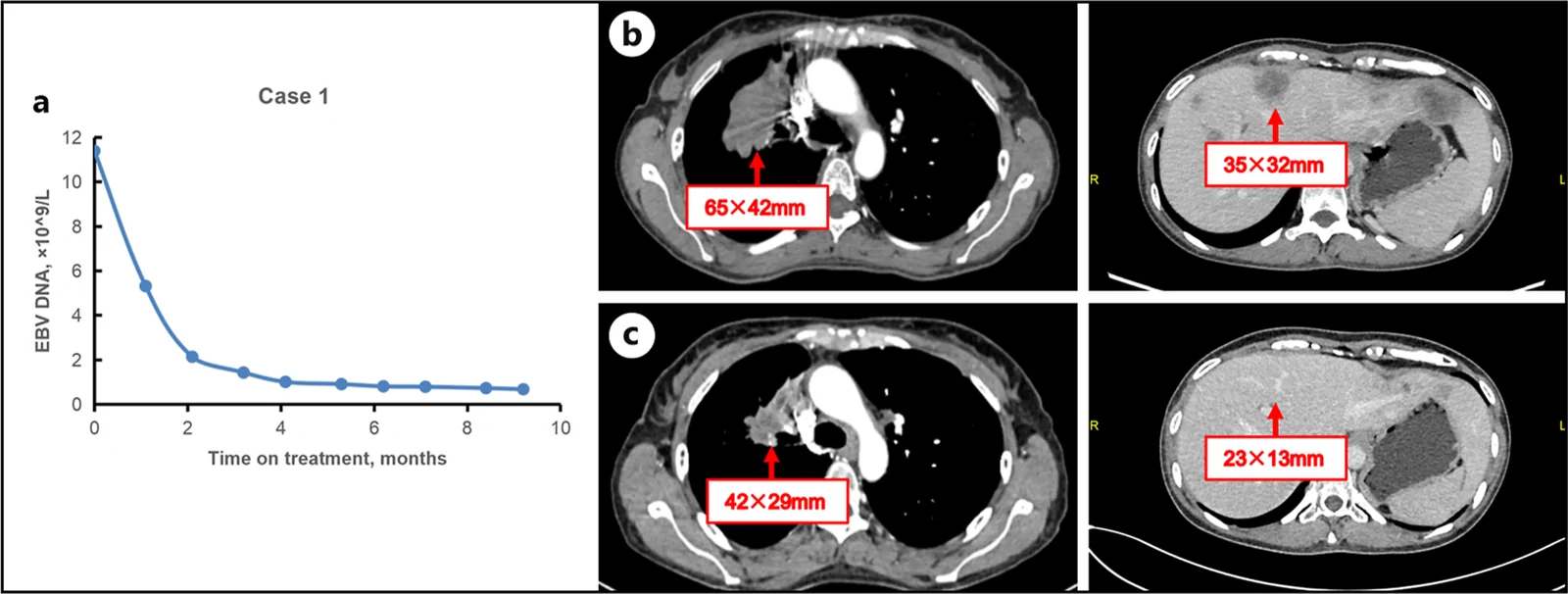
Changes in EBV DNA levels and tumor size in patient 1 with nasopharyngeal carcinoma and liver/lung metastases following treatment with utidelone plus PD-1 inhibitor. **a** Scatter plot illustrating the dynamics of plasma EBV DNA quantification. The last measurement before the first treatment cycle was taken as the baseline (time 0). The horizontal (x) axis indicates the time points from baseline, while the vertical (y) axis represents the quantified EBV DNA levels. **b** Representative baseline CT images of the lung and liver metastases. **c** Representative CT images of the same metastatic sites after 7 months of combination therapy with utidelone and camrelizumab.

### Case 2

A 57-year-old Chinese non-smoking man was diagnosed with pulmonary adenocarcinoma and multiple metastatic lesions including both lungs, adrenal glands, kidneys, lymph nodes, and brain in June 2021. Gene examination showed without sensitizing epidermal growth factor receptor (EGFR), anaplastic lymphoma kinase (ALK) and c-ros oncogene 1 (ROS1) mutations. Initially, he received sintilimab (PD-1 inhibitor) plus bevacizumab plus chemotherapy (pemetrexed and carboplatin) for 4 cycles, followed by 18 cycles of pemetrexed plus bevacizumab and sintilimab maintenance therapy. During first-line treatment, the total PFS was about 18 months. In December 2022, patient’s chest computed tomography (CT) suggested a progression of disease. He refused to receive paclitaxel concerning chemotherapy-induced hair loss. Thus, vinorelbine as second-line therapy was administered resulting in subsequent 4-month PFS. In May 2023, due to a significant elevation of tumor markers, he underwent third-line treatment with sintilimab plus anlotinib. Unfortunately, the tumor was not responsive to this regimen (Table 1).

In August 2023, the patient was admitted to Shenshan Medical Center of Sun Yat-sen memorial hospital and given a fourth-line therapy of UTD1 (30 mg/m^2^ once per day on days 1–5) plus sintilimab (200 mg once per day on day 1) of each cycle, once every 3 weeks. A follow-up CT scan after three cycles showed marked regression of the pulmonary mass and adrenal metastases, meeting the criteria for a PR (Fig. 2). No neurologic symptoms were noted at that time. However, after the fifth cycle in January 2024, the patient presented with new-onset right-sided ptosis and blurred vision. Subsequent brain magnetic resonance imaging revealed progression of the pre-existing brain metastases, while the extracranial disease remained controlled. The PFS from the start of UTD1 plus sintilimab to this brain progression was approximately 5 months, and the best overall response during the treatment course was PR (Table 1). During treatment, the patient developed grade 2 PN, with numbness in the fingers and toes that began after the third cycle. He received supportive treatment with mecobalamin and pregabalin, and the symptoms improved without requiring dose reduction or delay. No other significant adverse reactions were observed.

**Fig. 2.**
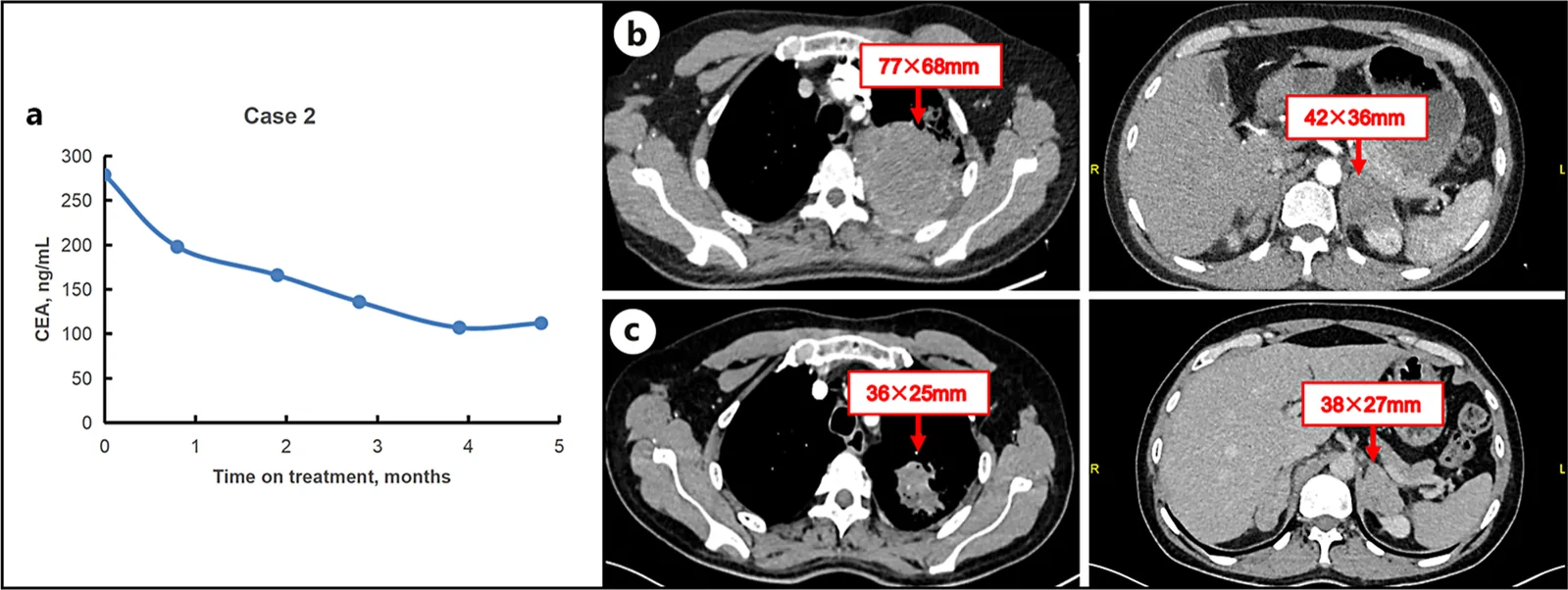
Changes in tumor marker CEA and tumor size in patient 2 with pulmonary adenocarcinoma following treatment with utidelone plus PD-1 inhibitor. **a** Scatter plot showing the quantitative changes in serum CEA levels over the course of treatment. The measurement taken immediately prior to the first treatment cycle was used as the baseline. **b** Representative baseline CT images demonstrating lung and adrenal metastases. **c** Representative CT images of the same metastatic sites after 4 months of combination therapy with utidelone and sintilimab.

### Case 3

The patient, a 65-year-old smoking Chinese man, was diagnosed with metastatic pulmonary adenocarcinoma without sensitive gene mutations in November 2022. In January 2023, he started to use sintilimab plus pemetrexed and carboplatin for six cycles, followed by additional six cycles of pemetrexed plus sintilimab as first-line therapy, achieving a PFS of approximately 9 months. In October 2023, patient’s chest CT suggested a progression of disease. He was treated with second-line treatment comprising nab-paclitaxel plus bevacizumab. After 8-cycle treatment, CT scan showed a progression disease (Table 1).

In April 2024, the patient was referred to Shenshan Medical Center of Sun Yat-sen memorial hospital and received UTD1 (30 mg/m2 days 1–5, every 3 weeks) in combination of camrelizumab (200 mg every 3 weeks) as the third-line treatment. He experienced a clinical improvement without any evidence of disease progression. After eight cycles, the antitumor effect was assessed as stable disease (Fig. 3). He developed numbness in the fingers and toes after the first cycle. UTD1 was discontinued due to grade 3 PN after eight cycles; neurologic symptoms persisted despite supportive treatment with mecobalamin, duloxetine, and pregabalin. His PN gradually resolved after UTD1 discontinuation. Subsequently, he received docetaxel, bevacizumab, and tislelizumab. A follow-up chest CT after one cycle of this subsequent regimen demonstrated progression of pulmonary metastases. The PFS from the start of UTD1 plus camrelizumab to disease progression was approximately 6 months (Table 1). In addition to PN, thrombocytopenia was observed, which improved after the administration of thrombopoietic growth factors.

**Fig. 3.**
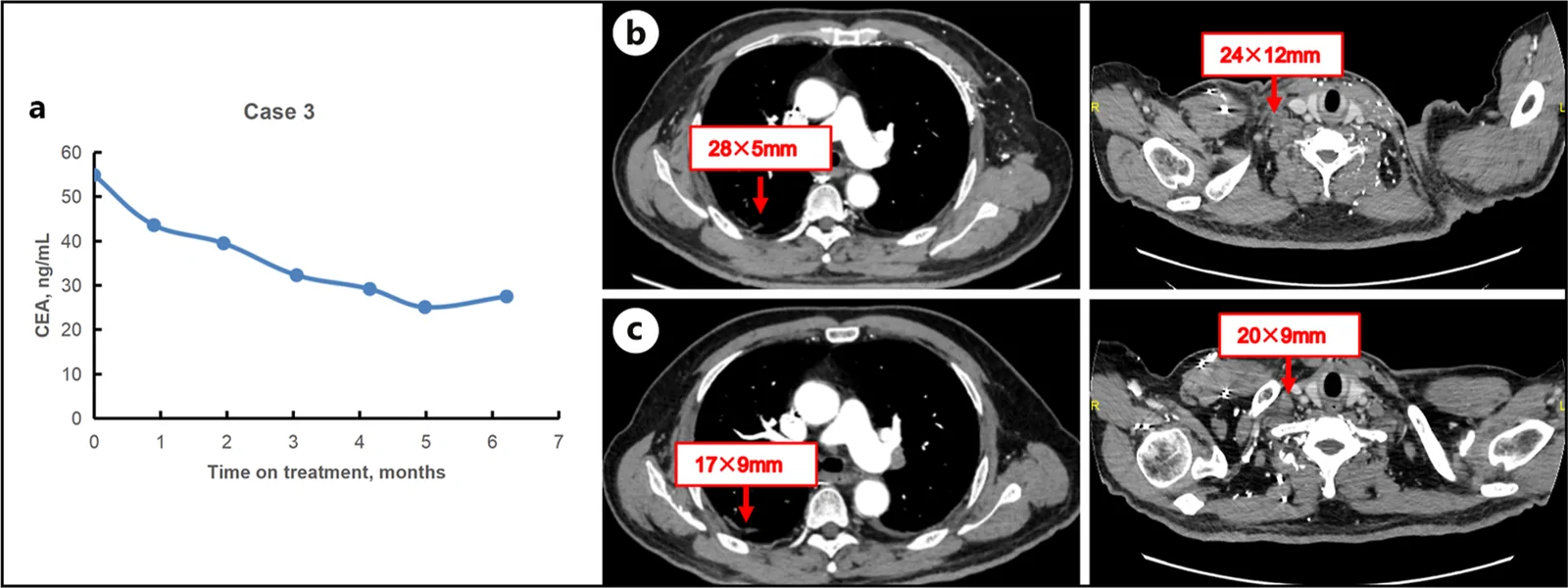
Changes in tumor marker CEA and tumor size in patient 3 with pulmonary adenocarcinoma following treatment with utidelone plus PD-1 inhibitor. **a** Scatter plot depicting the serial measurements of serum CEA levels over time. The value obtained immediately before initiation of the first treatment cycle was set as the baseline. **b** Representative baseline CT images showing lung and cervical lymph node metastases. **c** Representative CT images of lung and cervical lymph node metastases after 5 months of combination therapy with utidelone and sintilimab.

## Discussion

The antitumor activities of epothilones have been well characterized both in vitro and in vivo. The approvals of ixabepilone and UTD1 by FDA and NMPA, respectively, further validate their efficacy in treating metastatic or locally advanced breast cancer in patients who have previously failed treatments with anthracyclines, taxanes, and capecitabine [2, 14–18]. However, the severe adverse reactions and high cost associated with ixabepilone significantly restrict its clinical application [2–6], while UTD1 effectively mitigates these limitations. Currently, clinical research on UTD1 predominantly targets metastatic or locally advanced breast cancer that has not responded to prior anthracycline and taxane treatments. Phase II and III studies indicate that UTD1, whether administered as a monotherapy or in combination with capecitabine, can significantly enhance overall survival and PFS [5, 8, 9]. However, the efficacy of UTD1 in other tumor types, particularly in heavily pretreated advanced solid tumors after multiple lines of therapy, remains largely uncharacterized.

This case series has demonstrated the antitumor potential of UTD1 in heavily pretreated advanced solid tumors after multiple lines of therapy, which revealed promising outcomes for UTD1 plus PD-1 inhibitor regimens in 3 patients with different advanced solid tumor types (including nasopharyngeal carcinoma and lung adenocarcinoma) who developed resistance after multiple lines of treatment. Individual PFS times were 9 months (case 1), 5 months (case 2), and 6 months (case 3). Two patients achieved a PR and one achieved stable disease. Notably, cases 1 and 3, which had previously displayed resistance to taxanes, achieved PFS 9 months and 6 months, respectively.

Two patients with driver gene-negative lung adenocarcinoma (cases 2 and 3) demonstrated significant efficacy, achieving PFS of 5 and 6 months respectively, with the combination therapy with PD-1 inhibitor. A case involving a stage IV lung adenocarcinoma patient with an EGFR 19 deletion mutation, reported by Fang et al. [12], highlighted favorable outcome after receiving UTD1 combined with pembrolizumab after progression on third-line targeted therapy. This preliminary evidence indicates that patients with both driver gene-negative and driver gene-positive advanced lung adenocarcinoma may benefit from combined therapy involving UTD1 and PD-1 inhibitors after multiple lines of treatment. The individual PFS times observed in these heavily pretreated patients (9, 5, and 6 months) compare favorably with historical data on conventional chemotherapy in later-line treatments of non-small-cell lung cancer [19–21]. These findings underscore the clinical significance of UTD1 in later-line treatment of lung tumors.

The precise mechanisms by which UTD1 circumvents chemoresistance remain to be fully elucidated. The unique molecular structure of UTD1 may effectively overcome taxane resistance mechanisms by targeting distinct binding sites on tubulin. Although epothilones and taxanes exhibit analogous mechanisms of action by stabilizing microtubules, their distinct molecular architectures allow epothilones to retain efficacy against taxane-resistant tumor cell lines [15]. The property is similarly observed in other solid tumors beyond breast cancer, presenting a novel treatment option for patients with advanced tumors displaying taxane resistance. Additionally, previous studies have suggested that epothilones are not recognized as substrates by P-glycoprotein (P-gp, also known as MDR1) or breast cancer resistance protein (BCRP/ABCG2) [22, 23]. This characteristic may provide UTD1 with an advantage in circumventing drug resistance mechanisms. Notably, it has been reported that epothilones can inhibit the functions of the actin cytoskeleton and its key regulator, Rac1 GTPase [24], both of which play crucial roles in multidrug resistance (MDR) [25, 26].

We acknowledge the inability to precisely disentangle the individual anti-tumor contribution of utidelone or PD-1 inhibitor within the chemo-immunotherapy combination. Therefore, we next discuss the potential rationale for PD-1 rechallenge for these heavily pretreated patients. First, 3 patients in this study experienced no immune-related adverse events, which does not serve as a contraindication for PD-1 re-exposure. Second, numerous studies have demonstrated the potential activity of PD-(L)1 containing rechallenge strategies in patients previously treated with immunotherapy [27, 28]. UTD1 may help overcome resistance to PD-1/PD-L1 inhibitors by altering the immune microenvironment. Chemotherapy can induce immunogenic cell death, release tumor-specific antigens, reduce immunosuppressive factors in the tumor microenvironment, promote the activation and proliferation of immune cells, reshape the tumor microenvironment, and modulate immune responses [29]. Thus, chemotherapy may enhance the efficacy of immunotherapy and create more favorable conditions for subsequent treatment. Third, immunotherapy can in turn reverse chemotherapy resistance in tumor cells, thereby increasing their sensitivity to chemotherapy drugs [30]. Studies such as KEYNOTE-189 [31], IMpower150 [32], and IMpower133 [33] have confirmed that compared to chemotherapy alone, the combination of chemotherapy and immunotherapy can significantly improve treatment outcomes. Recent evidence from other multimodal immunotherapy-containing regimens further supports the potential clinical benefit of such combination strategies in heavily pretreated patients [34]. In summary, measurable disease control was achieved with UTD1 plus PD-1 inhibitors, suggesting potential immunotherapeutic responsiveness in this heavily pretreated population.

This case series identified that PN was the primary adverse event associated with UTD1, predominantly presented as numbness or pain in hands or feet related to sensory symptoms, and PN was the most common adverse event leading to treatment discontinuation, such as cases 1 and 3. Although the incidence of PN is high, it can be managed through prolonging interval, reducing dose, treatment interruption, or adjunctive symptomatic management. In a phase II trial conducted by Zhang and his colleagues, PN of grade 2 or 3 was reported in 29 participants. The median time of symptom resolution was observed to be 18 days. Furthermore, all individuals presenting with severe (grade 3) PN achieved a reduction in symptoms to grade 1 or lower after treatment discontinuation [5]. The phase III clinical study by Zhang et al. [8] also indicated that the median time from the onset of grade 3 PN to resolution to grade 1 or better was approximately 3–4 weeks, with about 96% of grade 3 PN resolving to grade 1 or better [9]. This indicates ideal remission rate and resolution time for UTD1-induced PN. Compared to other chemotherapeutic agents with similar mechanisms of action, particularly with ixabepilone, UTD1 showed some unique and important safety characteristics [2–4]. Notably, the incidences of neutropenia and other hematological toxicities caused by UTD1 was significantly lower [5, 8, 9], suggesting that, in contrast to paclitaxel and ixabepilone, UTD1 has a relatively mild impact on myelosuppression, and only 1 patient in this case series experienced transient thrombocytopenia. Furthermore, UTD1 did not appear to have significant effects on hepatic and renal function, and the gastrointestinal toxicity was also mild, consistent with the phase II and III findings of Zhang et al. [5, 8, 9]. In the study by Qiao et al. [11], the administration mode of UTD1 was changed from a 5-day intermittent infusion to a continuous 120-h intravenous infusion with total dose unchanged. Importantly, compared to the traditional administration mode, the continuous intravenous infusion of UTD1 demonstrated higher safety and tolerability. Further research is necessary to optimize the administration mode of UTD1 in order to minimize the occurrence of PN while maintaining efficacy. Given that PN led to treatment discontinuation in 2 of the 3 patients in this series, post-marketing pharmacovigilance studies of antineoplastic agents highlight the importance of continuous safety monitoring to identify and manage treatment-limiting toxicities in real-world settings [35].

Several important limitations of this case series should be acknowledged. First, the sample size is small (*n* = 3), which precludes any statistical inference and limits the generalizability of the findings. Second, the 3 cases encompass different tumor types (nasopharyngeal carcinoma and lung adenocarcinoma), different treatment lines (ranging from third-line to sixth-line), different PD-1 inhibitors (camrelizumab and sintilimab), and heterogeneous prior therapy histories. These sources of heterogeneity may confound the assessment of efficacy and safety attributable to the UTD1 plus PD-1 inhibitor. Third, given the retrospective, observational design, selection bias cannot be excluded. Finally, as treatment in cases 1 and 3 was discontinued due to toxicity rather than progression, the true PFS may be underestimated or overestimated. These observations should therefore be regarded as hypothesis generating, and confirmation in larger, prospective cohorts is warranted.

In conclusion, our case series demonstrated promising antitumor activity of UTD1 plus PD-1 inhibitors in patients with advanced or metastatic solid tumors after failure of multiple lines of therapy, resulting in clinically meaningful PFS and a manageable safety profile. This favorable benefit-risk profile suggests that UTD1 plus PD-1 inhibitors may represent a novel later-line therapeutic option in the arsenal of cancer treatment, particularly for tumors resistant to multiple drugs.

## Statement of Ethics

This study was conducted in accordance with the ethical principles outlined in the World Medical Association Declaration of Helsinki. The protocol was reviewed and approved by the Medical Ethics Committee of Shenshan Medical Center, Sun Yat-sen Memorial Hospital, Sun Yat-sen University (Approval No. 2025-SSKY-080-01). Written informed consent for publication of this case series was obtained from the legally authorized representatives (next of kin) of the deceased patients and from the living patients themselves.

## Conflict of Interest Statement

The authors have no relevant affiliations or financial involvement with any organization or entity with a financial interest in or financial conflict with the subject matter or materials discussed in the manuscript. This includes employment, consultancies, honoraria, stock ownership or options, expert testimony, grants or patents received or pending, or royalties.

## Funding Sources

This manuscript was granted by State Key Laboratory of Neurology and Oncology Drug Development (SKLSIM-F-2024138) and Beijing Xisike Clinical Oncology Research Foundation (Y-HR2020QN-0686) in the study design, execution, writing, and decision to publish.

## Author Contributions

Lisha Wu: investigation, resources, writing – original draft, writing – review and editing, visualization, and supervision; Huisheng Chen: investigation, writing – original draft, writing – review and editing, and visualization; Zihang Lin: writing – review and editing; Haiwei Xiao: resources, conceptualization, writing – review and editing; and Tao Qin: resources, conceptualization, writing – review and editing, supervision, and project administration.

## Data Availability

The data that support the findings of this study are not publicly available due to privacy reasons but are available from the corresponding author upon request.
